# Obesity reversibly depletes the basal cell population and enhances mammary epithelial cell estrogen receptor alpha expression and progenitor activity

**DOI:** 10.1186/s13058-017-0921-7

**Published:** 2017-11-29

**Authors:** Tamara Chamberlin, Joseph V. D’Amato, Lisa M. Arendt

**Affiliations:** 1 Program in Cellular and Molecular Biology, University of Wisconsin-Madison, 1525 Linden Drive, Madison, WI 53706 USA; 2 Department of Comparative Biosciences, School of Veterinary Medicine, University Wisconsin-Madison, 2015 Linden Drive, Rm 4354A, Madison, WI 53706 USA

**Keywords:** Breast, Mammary, Progenitor cells, Obesity, Estrogen receptor alpha

## Abstract

**Background:**

Obesity is correlated with an increased risk for developing postmenopausal breast cancer. Since obesity rates continue to rise worldwide, it is important to understand how the obese microenvironment influences normal mammary tissue to increase breast cancer risk. We hypothesized that obesity increases the proportion of luminal progenitor cells, which are thought to be the cells of origin for the most common types of breast cancer, potentially leading to an increased risk for breast cancer.

**Methods:**

To study the obese microenvironment within the mammary gland, we used a high-fat diet mouse model of obesity and human breast tissue from reduction mammoplasty surgery. We identified changes in breast epithelial cell populations using flow cytometry for cell surface markers, in vitro functional assays and expression of markers on breast tissue sections.

**Results:**

In both obese female mice and women, mammary epithelial cell populations demonstrated significant decreases in basal/myoepithelial cells, using either flow cytometry or cell-type-specific markers (SMA and p63). Estrogen receptor alpha (ERα) expression was significantly increased in luminal cells in obese mammary tissue, compared with control mice or breast tissue from lean women. Functional assays demonstrated significantly enhanced mammary epithelial progenitor activity in obese mammary epithelial cells and elevated numbers of ERα-positive epithelial cells that were co-labeled with markers of proliferation. Weight loss in a group of obese mice reversed increases in progenitor activity and ERα expression observed in obese mammary tissue.

**Conclusions:**

Obesity enhances ERα-positive epithelial cells, reduces the number of basal/myoepithelial cells, and increases stem/progenitor activity within normal mammary tissue in both women and female mice. These changes in epithelial cell populations induced by obesity are reversible with weight loss. Our findings support further studies to examine how obesity-induced changes in stem/progenitor cells impact breast tumor incidence and histologic tumor types.

**Electronic supplementary material:**

The online version of this article (doi:10.1186/s13058-017-0921-7) contains supplementary material, which is available to authorized users.

## Background

The World Health Organization estimates that more than 1.9 billion people are overweight, with 600 million people classified as obese [1]. Worldwide, the proportion of individuals with a body mass index (BMI) ≥25 kg/m^2^, which includes overweight and obese individuals, has risen to encompass 38% of women and 22.6% of girls [2]. Obesity has been shown to increase the risk of several different types of cancer in women, including postmenopausal breast cancer [3]. Weight gain following menopause significantly increases breast cancer risk and is correlated with poor prognosis in patients with breast cancer [4–8]. In contrast, obesity in premenopausal women has been shown in multiple studies to mildly reduce the risk of breast cancer [9–11]. The underlying mechanism for this divergence in breast cancer risk before and after menopause is not well-understood.

Mammary epithelium is composed of luminal cells, which express receptors to respond to ovarian hormones, including estrogen receptor alpha (ERα), and basal/myoepithelial cells, which are contractile and contact the basement membrane. Epithelial cells in both the luminal and basal lineages are derived from stem/progenitor cells. These stem/progenitor cells can be enriched in cell fractions that are characterized by their relative expression of cell surface markers, and their activity can be quantified by in vitro assays (for reviews see [12–16]). Current hypotheses suggest that the risk of developing breast cancer during a woman’s lifetime may be determined in part by the number of breast stem/progenitor cells that can serve as targets for transformation [17]. Recent studies using transgenic mouse models and human tissue xenografts have suggested that the most common types of breast cancer arise from mutations in progenitor cells in the luminal lineage [18–20]. In contrast, cells of the basal/myoepithelial lineage may have a critical tumor suppressive role in preventing the progression of ductal carcinoma in situ (DCIS) to invasive ductal carcinoma (for review, [21–23]). Understanding how obesity alters the complement of stem/progenitor cells within the mammary gland may provide insight into how obesity modulates breast cancer risk.

Data from multiple large epidemiological studies have demonstrated that postmenopausal women have an increased risk of developing ERα^+^ luminal breast tumors [24–26]. Postmenopausal women with higher BMI are more likely to develop tumors of the luminal type B molecular subtype, which are characterized by ERα expression, higher proliferative rates and reduced relapse-free survival compared to the luminal A subtype [27, 28]. However, obese premenopausal and postmenopausal women also have an increased likelihood of being diagnosed with ERα^-^ tumors compared with lean women [29, 30]. Here, we investigate changes in the mammary epithelium in both mice and humans to understand how obesity might impact the normal breast epithelium. We show that the timing of obesity and weight loss impact breast epithelial cell populations, which may lead to alterations in lifetime risk of breast cancer.

## Methods

### Primary human tissue

All human breast tissue procurement for these experiments was in compliance with the laws and institutional guidelines, as approved by the Institutional Review Board (IRB) committee from the University of Wisconsin-Madison. Disease-free, de-identified breast tissues were obtained from patients undergoing elective reduction mammoplasty with informed consent through the Translational Science BioCore BioBank at the Carbone Cancer Center at the University of Wisconsin-Madison. This research study was approved by the IRB as “Not Human Subject Research” with a limited patient dataset including patient age, date of service, and BMI. Tissue samples from patients aged 18–45 years were included in these studies.

### Mouse studies

All animal procedures were conducted in accordance with a protocol approved by the University of Wisconsin-Madison Institutional Animal Care and Use Committee. FVB/N (001800) and C57Bl/6 (000664) female mice were purchased from Jackson Laboratories and housed and handled in accordance with the Guide for Care and Use of Laboratory Animals in AAALAC-accredited facilities. All mice were given food and water *ad libitum*. For obesity studies, 3-week-old or 8-week-old mice were fed either low-fat maintenance chow (5% kcal from fat; Teklad Global #2018) or a high-fat diet (60% kcal from fat; Test Diet #58126) for 16–17 weeks. For weight loss studies, 8-week-old C57Bl/6 female mice were fed the high-fat diet for 15 weeks, then switched to the control diet for 5 weeks. Weight gain was calculated by subtracting the weight on the day of diet initiation from the weight measured each week. One hour prior to euthanasia, mice were injected with 200 mg/kg body weight 5-Bromo-2′-deoxyuridine (BrdU; Fisher Scientific, ICN10017101) diluted in PBS to label cells undergoing DNA synthesis. Thoracic and inguinal mammary glands were collected for analysis. The inguinal glands were utilized for histological assessment and whole mounts, and the remaining glands were collagenase-digested for stem/progenitor assays and flow cytometry.

To assess changes in the estrus cycle in response to obesity, vaginal cytology was performed daily for 14 days prior to tissue collection. Cells from the vaginal epithelium were flushed with 100 μl of PBS onto microscope slides (Fisher Scientific, 22-034979) and stained with Wright’s Stain (RICCA, 9350-16). The stage of the estrus cycle was determined using standard criteria with light microscopy [31]. Transitional smears were categorized based on the predominant feature on the slide [31]. Once the vaginal smears were collected, the slides were blinded to the treatment group and evaluated by a veterinary pathologist.

### Whole-mount preparation

The inguinal mammary glands were dissected from obese and lean mice and stretched onto microscope slides. The slides were immersed in 10% formalin overnight, defatted in xylenes for 24–48 h, and stained with carmine alum. The whole mounts were dehydrated in graded ethanol and stored in glycerol before analysis. Mammary branching was quantified by identifying branch points from the primary ducts originating from the nipple.

### Mammary epithelial cell isolation

Mouse mammary glands and human breast tissue were digested in organoid medium composed of DMEM/Ham’s F-12 (Corning, 10-090-CV) supplemented with 5 ng/ml human epidermal growth factor (EGF; Sigma, E9644), 10 μg/ml insulin (Sigma, I0516), 0.5 μg/ml hydrocortisone (Sigma, H0888), 5% calf serum, and 1% antibiotic/antimycotic solution (Corning, 30-004-CI) with 3 mg/ml collagenase A (Roche, 11088793001) and 100 U/ml hyaluronidase (Sigma, H3506) at 37 °C for either 1 hour (mouse) or overnight (human). Clusters of mammary epithelial cells, called organoids were allowed to settle at room temperature for 20 min, followed by centrifugation for 5 min at 8 × g relative centrifugal force. The supernatant was removed, the contaminating red blood cells lysed (ACK Lysing Buffer, Lonza, 10-548E), and the mammary organoids were washed with 5% calf serum in PBS. Human breast organoids were aliquoted and cryopreserved for future analysis. Mouse mammary organoids were dissociated to single cells following digestion for analysis in progenitor assays.

To isolate epithelial cells, mammary organoids were trypsinized by pipetting in 0.25% Trysin/EDTA (Corning, 25-053-CI). Cells were resuspended in organoid medium with 0.1 mg/ml DNAse (Roche 10104159001) and 5 mg/ml Dispase II (Roche, 04942078001) for 5 min. Single cells were isolated by filtration through a 40-μm cell strainer. Viable cells were counted using Trypan Blue (Corning, 25-900-CI) exclusion prior to further analysis.

### Flow cytometry

Flow cytometry of both human and mouse epithelial cells was performed and analyzed according to published guidelines [32]. Briefly, primary human breast tissue was dissociated to single cells, and endothelial, lymphocytic, monocytic, and fibroblastic lineages were depleted using antibodies to CD31 (MS-353), CD34 (180227), and CD45 (180367) from Thermo Scientific, fibroblast-specific protein IB10 (Sigma, F4771), and pan-mouse IgG Dynabeads (Dynal; Invitrogen, 115.31) according to the manufacturers’ instructions and as described previously [33]. Lineage-depleted single-cell suspensions or cells from immunomagnetic bead-sorted populations were resuspended at 1 × 10^6^ cells/ml in PBS containing 1% calf serum and bound with fluorescently conjugated antibodies to human EpCAM (12 ng/μl; APC; BD Biosciences, 347200) and CD49f (0.5 μg/μl; fluorescein isothiocyanate (FITC); BD Biosciences 555735). Primary mouse mammary epithelial cells were incubated with antibodies to detect CD49f (0.2 μg/μl; PerCP cy5.5; Biolegend, 313618) and EpCAM (0.2 μg/μl; APC; Biolegend, 118213). Endothelial, lymphocytic and monocytic lineages were depleted using antibodies specific to mouse CD31 (0.4 μg/μl; phycoerythrin (PE), 12-0311-83), CD45 (0.5 μg/μl; PE, 12-0451-81) and Ter119 (0.4 μg/μl; PE, 12-5921) from eBiosciences. All cells were incubated with primary antibodies for 20 min at 4 °C. To assess cell viability, all cells were stained with fixable viability dye eFluor 780 (eBiosciences, 65-0865) per manufacturers’ instructions. Antibody-bound cells were washed and resuspended at 1 × 10^6^ cells/ml and run on a BD LSR Fortessa (BD Biosciences). Gates were set on fluorescence minus one controls. Flow cytometry data were analyzed with the FlowJo software package (TreeStar).

### Progenitor assays

To assess mammosphere forming ability, 40,000 human mammary epithelial cells were plated in triplicate on 6-well ultra-low-attachment plates (Corning, CLS3473) in MEGM media (Lonza, cc-4136). For mice, 4000 mammary epithelial cells were plated in triplicate on 24-well ultra-low-attachment plates (Corning, CLS3473-24EA) in 500 μl EpiCult-B complete media (STEMCELL Technologies, 05610). Mammospheres formed for 7 days at 37 °C with 5% CO_2_ and were counted and imaged on an inverted microscope. To assess second-generation mammosphere formation, primary mammospheres were collected, trypsinized, and re-plated onto ultra-low-attachment plates for 7 days. Primary and secondary mammospheres were counted from 5 − 8 mice in each treatment group in triplicate.

To assess 3D colony formation, 40 μl of 1 mg/ml Type I rat tail collagen (Corning, 354236) was polymerized in 8-well chamber slides (Falcon, 354108); 1000 mouse mammary epithelial cells were resuspended in 500 μl organoid media containing 2% Matrigel (Corning, 354234) and layered over the collagen in duplicate. Colonies were formed for 7 days at 37 °C with 5% CO_2_ and were counted and imaged on an inverted microscope. Round and branching colonies were identified from five mice in each treatment group and quantified based on morphology.

To assess colony forming ability on tissue culture plates, viable cells from single-cell suspensions of lineage-depleted human mammary epithelial cells were plated at 10,000 cells/mL in 6-well plates in triplicate for adherent growth in MEGM (Lonza, cc-4136). Colonies were allowed to form for 7 days, after which non-adherent spheres (floating colonies) from adherent cultures were collected for analysis. Floating colonies were pelleted at 150 × g for 5 min and resuspended in 500 μl of growth medium and counted with an inverted light microscope.

To assess mouse mammary epithelial cell colony forming ability, 5000 epithelial cells were plated on 35-mm tissue culture plates (Cellstar, 627160) containing a feeder layer of 100,000 NIH 3 T3 cells (generously provided by Dr. Linda Schuler) treated with 20 mg/ml bleomycin sulfate (Millipore Calbiochem, 20-340) for 30 min. Epithelial cells were cultured in 2.5 ml of EpiCult-B medium supplemented with proliferation supplements (STEMCELL Technologies, 05610), 4 μg/ml heparin (STEMCELL Technologies, 07980), 0.02 μg/ml recombinant human basic fibroblast growth factor (STEMCELL Technologies, 02634), 0.01 μg/ml hEGF (Sigma, E9644) and 5% fetal bovine serum. After 24 h, the medium was changed to serum-free stem cell medium. Colonies were formed in duplicate for 7 days at 37 °C with 5% CO_2_.

Following colony formation, the plates were fixed with ice-cold methanol for 10 min and stored at -20 °C prior to analysis. To assess luminal and basal lineage markers, plates were immunohistochemically co-labeled for cytokeratin 8 (CK8) and CK14. Briefly, cells were permeabilized with 0.1% Triton X for 10 min and blocked for non-specific antibody binding using 1% BSA in PBS for 1 h. Cells were incubated with CK8 (Thermo Scientific, RM-2107-S0) at a 1:150 dilution in 1% BSA in PBS, followed by 30-min incubation with biotinylated goat anti-rabbit IgG (Vector, BA-1000) at 1:200 dilution in 1% BSA in PBS. Staining was visualized using VECTASTAIN ABC horseradish peroxidase (HRP) Kit (Vector, PK-4000) and VECTOR VIP Peroxidase (Vector, SK-4600). Cells were blocked with the avidin/biotin blocking kit (Vector, SP-2001). Cells were stained for CK14 (Thermo Scientific, MA5-11599) at a 1:150 dilution utilizing the MOM Kit (Vector BMK-2202) according to package instructions. CK14 staining was developed with 3,3-diaminobenzidine (DAB) Peroxidase (HRP) Substrate Kit (Vector, SK-4100). Colonies were counted from five to eight mice in each treatment group in duplicate using a light microscope.

### Immunohistochemical assessment

Mammary glands and breast tissue were fixed in 10% neutral buffered formalin for 18–24 h, embedded in paraffin and sectioned at 5 μm. Paraffin-embedded sections were rehydrated in graded alcohol and incubated with 0.5% H_2_O_2_ for 20 min. Sections underwent antigen retrieval in 0.1 M tris buffer (pH = 9.0) at 95 °C for 20 min. The tissue sections were blocked in 1% gelatin in TBST for 1 h and incubated with anti-mouse ERα antibodies (clone MC-20; Santa Cruz Biotechnology, sc-542) at a 1:500 dilution in 1% gelatin in TBST overnight at 4 °C. The sections were incubated with biotinylated goat anti-rabbit IgG secondary antibodies (Vector, BA-1000) at 1:500 dilution in 1% gelatin for 30 min, followed by VECTASTAIN ABC HRP Kit (Vector, PK-4000) for 30 min. The sections were developed with DAB Peroxidase (HRP) Substrate Kit (Vector, SK-4100), counterstained in hematoxylin, dehydrated in graded alcohol and mounted with Permount. A Nikon Eclipse 80 t microscope and SPOT camera were used for imaging the stained sections. The percentage of ERα^+^ cells were quantified by dividing the total number of ERα^+^ cells by the total number of epithelial cells within the ducts and multiplying by 100. Five images of each gland from five mice in each treatment group were analyzed.

### Immunofluorescence

Paraffin-embedded sections were rehydrated in graded alcohol and underwent antigen retrieval in 0.1 M tris buffer (pH = 9.0) or 0.01 M citrate buffer (pH = 6.0) at 95 °C for 20 min. Sections were blocked for 1 h and incubated with primary antibodies at 4 °C overnight. Primary antibodies included anti-mouse ERα (1:50, clone MC-20; Santa Cruz Biotechnology, sc-8002), anti-human ERα (1:200, clone F-10; Santa Cruz Biotechnology, sc-8002), anti-human CK14 (1:500; ThermoScientific, RB-9020), anti-human p63 that recognizes the C-terminal region of the protein (1:250, Santa Cruz Biotechnology, sc-6243), alpha smooth muscle actin (SMA, 1:5,000; Sigma, A5228), Ki67 (1:250; Abcam, 15580), or BrdU (clone BU1/75, 1:40; Accurate Chemical and Scientific Corporation, OBT0030S). Tissue sections were then incubated with secondary antibodies diluted 1:250 in 5% BSA in PBS for 30 min. Secondary antibodies were obtained from Life Technologies and included Alexa Fluor 546 goat anti-rabbit IgG (A11010), Alexa Fluor 488 goat anti-mouse IgG (A11001) and Alexa Fluor 488 goat anti-rat IgG (A11006). Sections were counterstained with 250 ng/ml 4′,6-diamidine-2′-phenylindole dihydrochloride (DAPI) for 5 min and mounted with the Slow-Fade mounting kit (Life Technologies, S2828).

The sections were imaged using a fluorescent microscope and the program NIS Elements BR 3.2. For basal/myoepithelial cell stains, including αSMA, p63, and CK14, sections were scored based on continuity of stain surrounding the luminal epithelial cells. The following score was used: 4 = 100%, 3 = 99–75%, 2 = 74–50%, and 1 = <50%. Five images were scored per tissue section. For mouse ERα and BrdU labeled samples, five images were quantified from seven mice per group. For human ERα and Ki67 labeling, ERα^+^, Ki67^+^, and co-labeled cells were quantified from three images per tissue sample. All cell counting and image alignment was performed using Image J software. To examine ERα expression in breast tissue from postmenopausal women, we utilized an available dataset of previously published data [34] from normal postmenopausal breast tissue from the Susan G. Komen Tissue Bank. We analyzed the correlation between ERα expression that was quantified as described [34] and the patients’ recorded BMI values.

### Statistical analyses

Results were expressed as the mean ± standard deviation (s.d.), unless stated otherwise. Statistical differences were determined using the Mann-Whitney test, unless stated otherwise. Correlation with BMI in humans was analyzed using two-tailed Pearson correlation. Investigators were blinded to the BMI data until analyses were complete. *P* values of 0.05 or less were considered to denote significance. Statistical analyses were conducted using GraphPad Prism 6 (GraphPad Software).

## Results

### Obesity alters mammary epithelial cell populations

To understand how obesity might alter normal breast tissue and lead to increased breast cancer risk in obese women, we utilized a high-fat diet model of obesity in mice. Intact C57Bl/6 female mice were fed either a control diet or a diet containing extra calories from fat (high-fat diet (HFD)). After 17 weeks, mice fed HFD weighed significantly more (33.5 g ± 9.0; *n* = 7) than control mice (24.5 g ± 3.2; *n* = 7; *p* = 0.004, Mann-Whitney test). In addition to increased body weight, there was significantly increased visceral fat surrounding the uterus in mice fed HFD (Additional file 1: Figure S1A). Within the mammary gland, adipocytes were significantly larger (Additional file 1: Figure S1B, C). Crown-like structures composed of F4/80^+^ macrophages that surround necrotic adipocytes have been observed previously in mammary glands in obesity [35–37]. These F4/80^+^ crown-like structures were found only in mammary glands from mice fed HFD (Additional file 1: Figure S1B, D), suggesting that the HFD induced obesity.

**Fig. 1:**
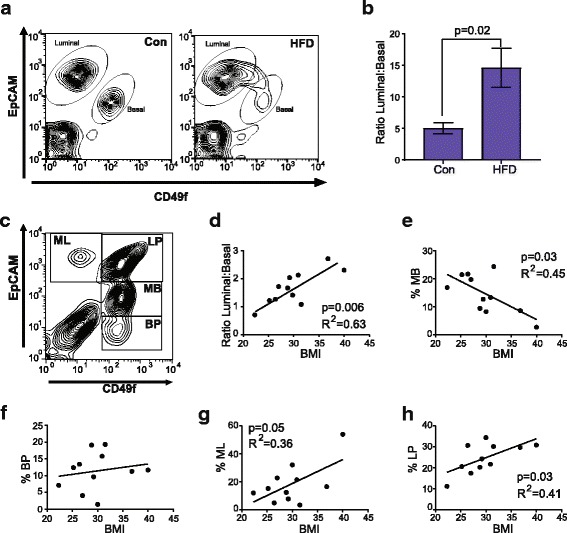
Obesity enhances luminal and decreases basal/myoepithelial cell populations. Mammary epithelial cells isolated from control (Con) and obese (high-fat diet (HFD)) C57Bl/6 mice were stained with EpCAM and CD49f and quantified using flow cytometry (**a**, **b**). **a** Representative contour plots depicting gates for luminal cells (EpCAM^hi^CD49f^lo^) and basal cells (EpCAM^lo^CD49f^hi^). The percentage of luminal and basal cells were quantified from total mammary-lineage-positive cells in three experiments (*n* = 9 mice/group). **b** Results were determined as a ratio of luminal to basal cells from obese mice compared with control mice and represented as mean ± s.d. Human breast epithelial cells were isolated from reduction mammoplasty samples, stained with EpCAM and CD49f, and quantified using flow cytometry (*n* = 11 patient samples). **c** Representative contour plot depicting gates for mature luminal (ML, EpCAM^hi^CD49f^-^), luminal progenitor (LP, EpCAM^hi^CD49f^+^), mature basal (MB, EpCAM^lo^CD49f^+^), and basal progenitor cells (BP, EpCAM^-^CD49f^+^). **d** Correlation scatter plot with best fit line representing the ratio of the percentage of luminal to basal/myoepithelial cells. The percentage of positive cells was the number of positive cells in the gate compared to the total number of mammary-lineage-positive cells. Correlation scatter plot with best fit line representing the percentage of MB (**e**), BP (**f**), ML (**g**), and LP (**h**) against body mass index (BMI)

To investigate how obesity perturbs the normal hierarchy of the mammary epithelium, we dissociated mammary glands isolated from control mice and mice fed HFD. Single cells were stained with antibodies for EpCAM and CD49f to detect mammary lineage cells, antibodies for CD31, CD45 and Ter119 conjugated with PE to identify stromal cell populations, and a live/dead fluorescent marker, and analyzed using flow cytometry according to guidelines (Additional file 2: Figure S2A) [32]. As described previously, staining EpCAM and CD49f separated the epithelial cells into two populations, luminal EpCAM^hi^CD49f^lo^ cells and basal EpCAM^lo^CD49f^hi^ cells [38–40]. In control mice, the epithelial cells were separated into two distinct populations (Fig. 1a). However, in mice fed HFD, the percentage of basal epithelial cells was significantly decreased, and this population was not distinctly separated from the luminal cell population as compared to the control mice (Fig. 1a). When compared to control mice, the ratio of luminal to basal epithelial cells was significantly increased in mice fed HFD (Fig. 1b). These results suggest that obesity enhances luminal cells and reduces basal cell populations in epithelial cells in murine mammary glands.

Since we observed a significant increase in the ratio of luminal to basal cells in obese mammary glands, we examined human breast tissue isolated from reduction mammoplasty surgery. This tissue was obtained with a limited dataset, including age and BMI. However, parity and stage of the menstrual cycle, which may also alter breast epithelial cell populations, were unknown for all donors of tissue samples. In human breast tissue, staining dissociated breast epithelial cells with markers EpCAM and CD49f has been shown to delineate four populations of epithelial cells [18, 19, 41, 42]: EpCAM^hi^CD49f^-^ mature luminal cells (ML), EpCAM^hi^CD49f^+^ luminal progenitor cells (LP), EpCAM^lo^CD49f^+^ mature basal cells (MB), and EpCAM^-^CD49f^+^ basal progenitor cells (BP; Fig. 1c). To assess these populations, dissociated cells were lineage-depleted using antibody-conjugated beads and stained to detect live cells and with antibodies for EpCAM and CD49f (Additional file 2: Figure S2B). Epithelial cells were quantified in each population and examined in the context of BMI. Similar to our observations in the mouse mammary glands, the ratio of luminal to basal/myoepithelial cells was significantly enhanced with increasing BMI (Fig. 1d). Increasing BMI correlated with reductions in the basal/myoepithelial cell population. However, this correlation was observed only within the MB population (Fig. 1e). The BP population was not significantly altered by BMI (Fig. 1f). In contrast, both the ML (Fig. 1g) and LP (Fig. 1h) cell populations were significantly enhanced with increasing BMI. These results suggest that similar to the murine mammary gland, human breast epithelial cell populations are altered in response to obesity, with increased luminal cell populations and decreased basal/myoepithelial cell populations.

### Obesity reduces basal/myoepithelial cells and enhances ERα-positive luminal cells

**Fig. 2:**
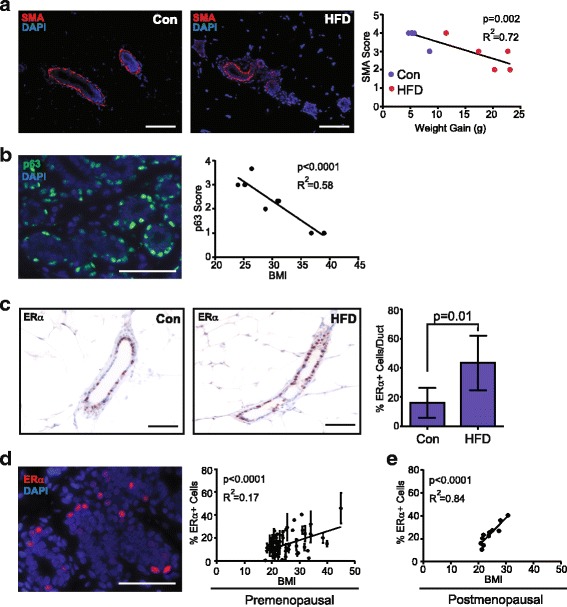
Obesity reduces basal/myoepithelial cells and enhances estrogen receptor (ER)α-positive luminal cells. **a** Basal/myoepithelial cells were detected using immunofluorescence (IF) for alpha smooth muscle actin (SMA, red) within the mammary glands of mice fed a control diet (Con) or high-fat diet (HFD). Nuclei were detected with 4′6-diamidine-2′-phenylidole dihydrochloride (DAPI) (blue). SMA continuity was scored as described in “Methods” and graphed in relation to weight gain. **b** p63^+^ (green) basal/myoepithelial cells were detected using IF within breast tissue from reduction mammoplasty surgery in women aged 20–45 years. Nuclei were detected with DAPI (blue). p63 expression was scored as described in “Methods” and graphed in relation to body mass index (BMI; n = 7). **c** ERα expression was immunohistochemically quantified within mammary glands from mice fed Con or HFD (5 mice/group; mean ± s.d.). **d** ERα expression (red) was detected using IF within human breast tissue from women ages 20–45 years. Nuclei were detected using DAPI (blue). ERα expression was plotted in relation to BMI (n = 39; mean ± s.d.). **e** ERα expression was plotted in relation to BMI, as reported through the Susan G. Komen Tissue Repository (n = 11). Magnification bar = 100 μm

To visually assess how changes in epithelial cell populations manifest within the mammary tissue architecture, we examined markers for luminal and basal cells in murine mammary tissue. To evaluate basal cells, we examined the expression of myoepithelial cell marker αSMA using immunofluorescence. In control glands, SMA was expressed in a continuous layer surrounding the ducts. In contrast, the glands of the mice fed HFD demonstrated discontinuous expression of SMA compared to the control mice, and this decreased expression of SMA was inversely correlated with increasing weight gain on the HFD (Fig. 2a). This observation is consistent with a previous study that demonstrated reduced SMA expression surrounding the ducts in mammary glands from obese mice [37]. These results suggest that basal cells were significantly reduced in obese mice compared to control mice.

To delineate basal/myoepithelial cells in normal breast tissue, we examined expression of CK14 and p63 in human reduction mammoplasty tissue. Within the lobules, we observed no significant changes in CK14 expression that correlated with BMI, although there was significant variability in total CK14 expression among patient samples (Additional file 2: Figure S2C). We and others have observed variability in the cell populations expressing CK14 [41, 43, 44], so we also examined expression of the basal/myoepithelial cell marker p63, a member of the p53 family of transcription factors [45]. Similar to CK14, alterations in p63 continuity were observed within the patient samples. However, when p63 continuity was examined in the context of BMI, p63 expression was significantly decreased with increasing BMI (Fig. 2b). These findings suggest that basal/myoepithelial cells may be decreased within mammary tissue with increasing obesity in both mice and humans.

Since we observed changes in the luminal to basal/myoepithelial cell ratios and luminal epithelial cell populations in human breast tissue using flow cytometry, we examined expression of the luminal cell marker ERα, which is enriched in the mature luminal cell population in mice and humans [18, 19, 41, 46, 47]. In mammary glands from obese mice, the percentage of ERα^+^ epithelial cells was significantly increased, compared to those from control mice (Fig. 2c). Within breast tissue from premenopausal women, we observed variability in ERα expression (Fig. 2d). However, when examined within the context of BMI, ERα expression was significantly and positively correlated with increasing BMI (Fig. 2d).

Steroid hormone receptor expression levels, including ERα, fluctuate over the course of the menstrual cycle [48, 49]. Given that we did not have patient information on the timing of the menstrual cycle during breast tissue collection, hormonal changes may have contributed to the variability in ERα expression we observed in our normal tissue samples. To address this question, we examined ERα expression in breast tissue from postmenopausal women using an available dataset of characterized normal postmenopausal breast tissue from the Susan G. Komen Tissue Bank [34]. In postmenopausal women, ERα expression was significantly correlated with increasing BMI (Fig. 2e), with significantly less variability than we observed in our group of premenopausal women. Together, these findings suggest obesity significantly impacts normal mouse mammary and human breast tissue by reducing basal/myoepithelial cells and enhancing ERα^+^ epithelial cells.

### HFD feeding during puberty reduces mammary ductal branching

**Fig. 3:**
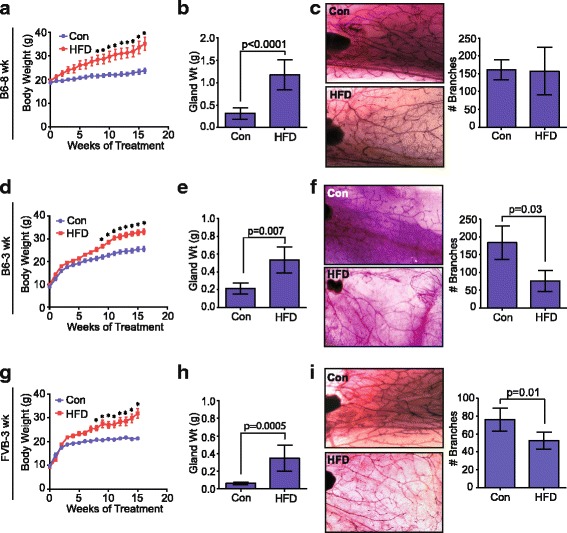
High-fat diet (HFD) feeding during puberty reduces ductal branching. **a** C57Bl/6 female mice were fed a HFD or control diet (Con) starting at 8 weeks of age (B6-8 wk). Weight was determined weekly for 17 weeks (n = 7 mice/group; mean ± s.e.m.). Significance was determined using two way analysis of variance (ANOVA) (*p < 0.05). **b** Weights (Wt) of inguinal mammary glands from Con and HFD-fed B6-8 wk mice after 17 weeks on the diets (n = 7 mice/group). **c** Mammary gland whole mounts of inguinal mammary glands from Con and HFD-fed B6-8 wk mice. Tertiary branches were counted in both groups (n = 5 mice/group). **d** C57BL/6 female mice were fed HFD or Con starting at 3 weeks of age (B6-3 wk) for 17 weeks (n = 9 mice/group; mean ± s.e.m.). Significance was determined using two way ANOVA (*p < 0.05). **e** After 17 weeks, inguinal mammary glands were weighed from B6-3 wk mice (n = 9 mice/group). **f** Mammary gland whole mounts of inguinal mammary glands from B6-3 wk mice. Tertiary branches were counted in both groups (n = 5 mice/group). **g** FVB/N female mice were fed a HFD or Con starting at 3 weeks of age (FVB-3 wk) for 17 weeks (n = 9 mice/group; mean ± s.e.m.). Significance was determined using two way ANOVA (*p < 0.05). **h** After 17 weeks, inguinal mammary glands were weighed from FVB-3 wk mice (n = 9 mice/group). **i** Mammary gland whole mounts of inguinal mammary glands from Con and HFD-fed FVB 3-wk mice. Tertiary branches were counted in both groups (n = 5 mice/group). Bars represent mean ± s.d. Magnification of images × 10

Given the importance of ERα expression in normal mammary gland development, we hypothesized that feeding mice with a HFD during puberty may result in developmental changes in growth of the mammary gland. First, we examined mammary gland structure in mice when the HFD was started after the completion of puberty. In C57Bl/6 female mice fed a HFD starting at 8 weeks of age (B6-8 wk in Fig. 3a), total body weight was significantly increased within 8 weeks, and mice gained a significant amount of weight due to the diet treatment compared to control mice (Fig. 3a, Additional file 3: Figure S3A). After 17 weeks on the HFD, the inguinal mammary glands were significantly larger than those from control-diet-fed mice (Fig. 3b). In contrast, female FVB/N mice fed the HFD for 14 weeks starting at 8 weeks of age (FVB-8 wk in Fig. 3g) did not significantly change total body weight or gain significant weight compared to control mice (Additional file 3: Figure S3B, S3C). We also observed no significant differences in inguinal gland weight in the mice fed HFD compared to controls (Additional file 3: Figure S3D), and no crown-like structures were observed within the mammary adipose tissue in either control diet or HFD-fed mice (data not shown). This is consistent with a previous report suggesting that FVB/N mice are more resistant to weight gain [50].

To examine global changes in the structure of the mammary glands, mammary whole mounts of the inguinal glands were examined. No significant differences in mammary gland morphology and branching were observed in the mammary glands from HFD-fed B6 8-week-old mice compared to control mice (Fig. 3c). Consistent with the limited weight gain in the HFD-fed FVB 8-week-old mice, there were also no differences observed in mammary gland branching between the HFD and control diet-fed mice (Additional file 3: Figure S3E).

To look at the effects of a HFD on mammary gland development, both C57Bl/6 and FVB/N mice were started on either the HFD or control diet after weaning at 3 weeks of age (B6-3 wk and FVB-3 wk, respectively, on Fig. 3). In both strains of mice, HFD-fed mice had greater body weight (Fig. 3d, g) and weight gain (Additional file 3: Figure S3F, J) following a period of total body growth during puberty. In addition to increased body weight, visceral fat surrounding the uterus was significantly increased in both groups of HFD-fed mice (Additional file 3: Figure S3G, K). The weight gain in HFD-fed FVB 3-week-old mice is in contrast to the limited weight gain observed in FVB/N mice fed the HFD starting at 8 weeks of age (Additional file 3: Figure S3B, C). This suggests that the age of feeding with the HFD impacts total weight gain in this strain of mice. In both FVB/N and C57Bl/6 strains of mice, inguinal mammary glands from the HFD-fed B6 3-week-old and FVB 3-week-old mice weighed significantly more than those from control mice after 17 weeks on the diets (Fig. 3e, h). Compared to control mice, we also observed significant increases in adipocyte diameters within the inguinal glands (Additional file 3: Figure S3H, L), and the presence of crown-like structures (Additional file 3: Figure S3I, M) in mammary glands of HFD-fed B6 3-week-old and FVB 3-week-old mice. In contrast to the mammary glands from HFD-fed B6 8-week-old mice, mammary whole mounts from both HFD-fed B6 3-week-old and FVB 3-week-old mice demonstrated significantly reduced tertiary branching compared to those from the control mice (Fig. 3f, i). Together, these results suggest that a HFD during puberty significantly impedes mammary gland development by reducing ductal growth.

Since the mammary tree undergoes cyclic remodeling over the course of the estrus cycle, with increased branching in response to changes in ovarian hormones, we examined how obesity altered the estrus cycle of the mice on each diet. To examine changes in the estrus cycle, we examined vaginal cytology from C57Bl/6 and FVB/N mice to determine the number of days spent in each stage of the estrus cycle. No significant differences were observed between the control and obese mice in either mouse strain (Additional file 4: Figure S4A, B). We did observe one mouse in the C57Bl/6 HFD group that demonstrated persistent diestrus, which may have resulted due to the sampling method for vaginal cytology [31]. The uterus is also very sensitive to the effects of ovarian steroids, and uterine weight is frequently used to assess increased exposure to circulating estrogens [51]. To assess long-term changes in ovarian steroids in response to obesity, we examined uterine weights. No significant differences in uterine weights were observed between control and obese mice within either strain of mice (Additional file 4: Figure S4C, D). Together, these results suggest that obesity did not induce long-term changes in ovarian cycling that may lead to changes in ductal branching observed in our HFD-fed 3-week groups.

Given the differences in ductal branching, we next investigated how HFD feeding started at 3 weeks of age altered mammary epithelial cell populations. Mammary glands were isolated from HFD-fed B6 3-week-old or control mice. Similar to our observations in B6 8-week-old mice, HFD-fed B6 3-week-old mice had a significantly increased ratio of luminal to basal epithelial cells compared with control mice (Additional file 5: Figure S5A). Also similar to the HFD-fed B6 8-week-old mice, both HFD-fed B6 3-week-old and FVB 3-week-old mice had significantly reduced SMA expression surrounding ducts (Additional file 5: Figure S5B), and significantly increased epithelial ERα expression compared with control glands (Additional file 5: Figure S5C). These results suggest that the changes in epithelial cell populations within the HFD-fed B6 3-week-old and FVB 3-week-old mice are consistent with those from HFD-fed B6 8-week-old mice regardless of the timing of induction of obesity. Additionally, these changes in epithelial cell populations are not mouse strain-specific. Given that HFD-fed FVB 8-week-old mice did not gain significant weight, we examined mammary tissue to assess changes in SMA and ERα expression. No significant differences were observed between HFD-fed and control mice in expression of either SMA or ERα (Additional file 5: Figure S5D, E), suggesting that the epithelial cell population changes observed in obese mice were due to weight gain rather than the long-term HFD.

### Obesity enhances mammary epithelial cell progenitor activity

**Fig. 4:**
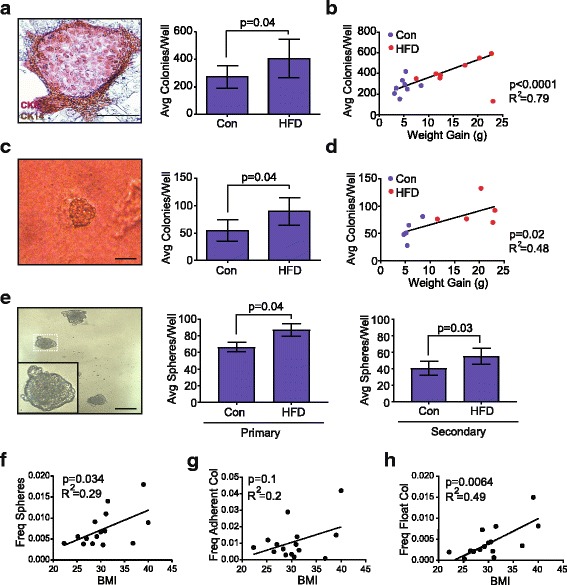
Obesity enhances mammary epithelial cell progenitor activity. **a** Mammary epithelial cell colony stained for cytokeratin (CK)8 (purple) and CK14 (brown) grown on an NIH 3T3 cell feeder layer. Colonies were quantified from *n* = 8 mice/group in duplicate. **b** Correlation scatterplot with best fit line representing the average (Avg) number of mammary epithelial progenitor colonies per well with respect to mouse weight gain (*n* = 16 mice). **c** Mammary epithelial cell round colony formation on collagen gel. Colonies were quantified from five mice per group, in duplicate. **d** Correlation scatterplot with best fit line depicting the average number of mammary epithelial round colonies on collagen gels with mouse weight gain (*n* = 10 mice). **e** Mammosphere formation in suspension on ultra-low adherence plates. Primary and secondary generation mammospheres were quantified from eight mice per group in triplicate. **f** Correlation scatterplot with best fit line representing frequency of mammosphere formation from breast epithelial cells isolated from reduction mammoplasty tissue with respect to body mass index (BMI) quantified from 15 patient samples in triplicate. **g** Correlation scatterplot with best fit line representing average adherent colony formation from 15 patient samples in triplicate with respect to BMI. **h** Correlation scatterplot with best fit line representing average floating colony formation from 15 patient samples in triplicate with respect to BMI. Bars represent mean ± s.d. Magnification bar = 100 μm. Freq, frequency; HFD, high-fat diet; Con, Control diet

Since obesity alters populations of cells both in the luminal and basal/myoepithelial mammary epithelial cell lineages, we hypothesized that epithelial progenitor activity might also be changed. To assess mammary epithelial progenitor activity, we enzymatically dissociated epithelial cells from the glands of lean and obese C57Bl/6 adult mice and plated the cells in vitro in colony-forming assays using limiting dilutions established from pilot studies. When plated on a feeder layer of NIH 3 T3 cells in limiting dilution, mammary epithelial cells formed colonies that expressed the luminal marker CK8 and the basal marker CK14 (Fig. 4a). The ability to form CK8/14^+^ colonies was significantly enhanced in epithelial cells isolated from glands in the HFD-fed compared to the controls (Fig. 4a). Since the obese mice exhibited some variability in weight gain, we also examined the correlation between colony formation and total weight gained on each diet. CK8/14^+^ colony-forming ability was strongly correlated with increased weight gain (Fig. 4b).

In human epithelial progenitor assays, epithelial cells with the ability to form round colonies on collagen gels were enriched in the EpCAM^hi^ epithelial cell population, while epithelial cells with the ability to form duct-like structures were enriched in the EpCAM^lo/-^ basal/myoepithelial populations [19, 41]. To assess the ability of the mouse epithelial cells to form round and ductal colonies, we plated dissociated epithelial cells in limiting dilution on collagen gels. No significant differences were observed in the formation of ductal colonies in epithelial cells from the HFD and control mice (data not shown). However, round colonies were significantly increased in epithelial cells from mice fed HFD compared to control mice when grown on the collagen gels (Fig. 4c). This increase in round colonies was also significantly correlated with increasing weight gain on the respective diets (Fig. 4d).

Total progenitor activity has also been assessed by the ability of epithelial cells to grow in suspension and form colonies called mammospheres [52]. Mouse mammary epithelial cells were isolated and plated in limiting dilution on ultra-low attachment plates and suspended colonies were counted after 7 days. Following mammosphere formation, the colonies were then dissociated and plated again to form secondary colonies. Epithelial cells from obese mice generated significantly increased numbers of primary and secondary mammospheres compared with those from control mice (Fig. 4e). Together, these results suggest that obesity enhances epithelial progenitor activity in the mammary glands of obese mice compared to control mice.

Since we observed alterations in epithelial cell surface markers in human breast reduction mammoplasty samples, we hypothesized that obesity would also increase progenitor activity with increasing BMI. To test this hypothesis, we isolated breast epithelial cells from reduction mammoplasty tissue from women with known BMI, and plated cells in limiting dilution in mammosphere assays and for colony formation on adherent plates. Mammosphere formation was significantly correlated with increasing BMI (Fig. 4f). Adherent colonies, which express luminal (CK8), basal (CK14) or mixed CK8/14^+^ markers, form in colony-forming assays with human epithelial cells [19, 33, 41, 42, 53]. In addition, floating colonies, which express only luminal markers and are thought to form from luminal-restricted progenitor cells, form in suspension over the adherent plates [19, 33, 41]. We quantified both adherent colonies and floating colonies and examined the relationship of these colonies to BMI. Adherent colonies, which are thought to originate from basal and luminal progenitor cells [54], were not significantly correlated with increasing BMI (Fig. 4g). However, floating colony formation was significantly enhanced in patient samples with increasing BMI (Fig. 4h), suggesting that luminal progenitor cells were significantly enhanced in human samples with increasing BMI. Together, these results suggest that increasing obesity enhances progenitor activity in both mouse and human mammary tissue.

### ERα-positive cells co-label with proliferation markers

**Fig. 5:**
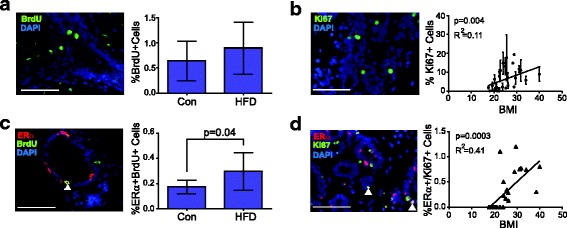
Estrogen receptor (ER)α co-localization with proliferation markers is enhanced in obese tissue. **a** Proliferating cells were quantified in mammary epithelial cells in tissue from adult C57Bl/6 mice fed a control diet (Con) or high fat diet (HFD) using immunofluorescence for 5-bromo-2′-deoxyuridine (BrdU) in the mammary glands (n = 7 mice/group). Five images were quantified per × 400 field/mouse. **b** Immunofluorescence for Ki67 expression in breast epithelial cells from reduction mammoplasty tissue. Correlation scatterplot with best fit line demonstrating the average percentage of Ki67-positive cells per × 400 field, against body mass index (BMI). Three images/lobule were analyzed for each sample (n = 28). **c** Immunofluorescence for BrdU and ERα in mammary glands from mice fed Con or HFD (n = 7 mice/group). Five images were quantified per × 400 field/mouse. **d** Immunofluorescence for Ki67 and ERα in breast epithelial cells from reduction mammoplasty tissue. Correlation scatterplot with best fit line depicting the percentage of cells that co-label with antibodies for Ki67 and ERα per × 400 field with respect to BMI. Three images/lobule were analyzed for each sample (n = 27). Magnification bar = 100 μm

Given the enhanced mammary epithelial progenitor activity that we observed from cells isolated from the obese microenvironment, and the expansion of the luminal population, we investigated the proliferation rates of epithelial cells using immunofluorescence. One hour prior to euthanasia, lean and obese mice were injected with BrdU to label cells in the S-phase of the cell cycle. No significant differences were observed in the proliferation rates of epithelial cells between mammary glands from obese and lean mice (Fig. 5a). To assess proliferation in human breast epithelial cells, we examined Ki67 expression (Fig. 5b). The percentage of Ki67^+^ cells was variable among patient breast samples and within lobules of the same patient sample. Interestingly, even with this variability of expression, we observed significant positive correlation with increasing BMI (Fig. 5b). Since the phase of both the estrus and menstrual cycles change the proliferation rates of mammary epithelial cells in mice and humans [55–57], the variability in circulating hormones in our samples may have contributed to the variability in proliferation that we observed.

In both humans and mice, rare ERα^+^ cells have been identified that co-label with markers of proliferation [58–60]. Since we observed a significant increase in ERα^+^ cells within mammary glands from obese mice, we examined the co-localization of ERα with BrdU in murine epithelial cells. Consistent with previous reports [58–61], mammary glands from control mice had a low frequency of ERα^+^BrdU^+^ cells (Fig. 5c). The percentage of ERα^+^BrdU^+^ cells was significantly increased in the mammary glands of obese mice compared with control mice (Fig. 5c). In human breast tissue, we also identified epithelial cells labeled with both ERα and Ki67 (Fig. 5d). Interestingly, these cells were not detected in patient samples derived from women with BMI <25, which corresponds with the normal BMI range. However, these ERα^+^Ki67^+^ cells were observed in breast samples from women in both the overweight (BMI 25–29.9) and obese (BMI >30) ranges. The percentage of ERα^+^Ki67^+^ cells was significantly correlated with increasing BMI (Fig. 5d). Together these results suggest that obesity enhances a rare population of ERα^+^ cells with the ability to proliferate.

### Weight loss reverses changes in mammary progenitor activity

**Fig. 6:**
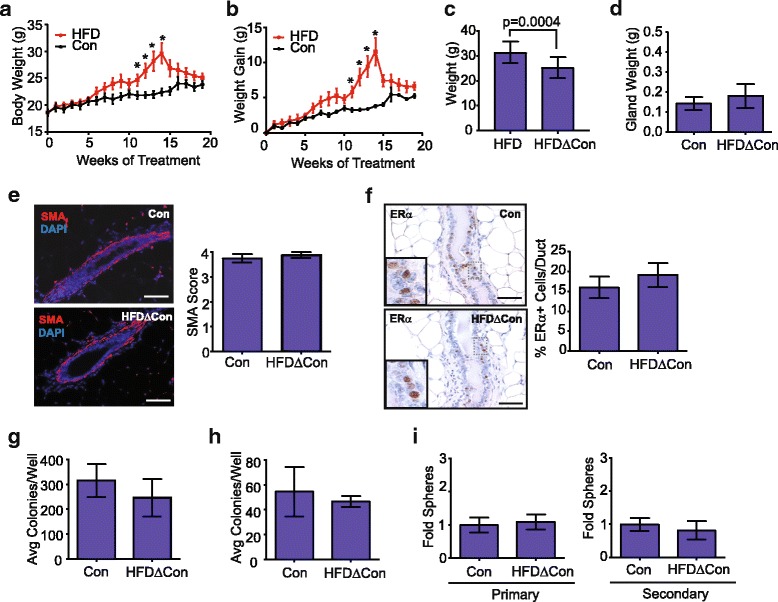
Weight loss normalizes epithelial cell populations and progenitor activity. Body weight changes (**a**) and weight gain (**b**) in 8-week-old C57BL/6 female mice fed a high-fat diet (HFD) for 15 weeks and then changed to the control diet (HFDΔCon; *n* = 5 mice) for 5 weeks or were fed the control diet (Con) for 20 weeks. (*n* = 5 mice; mean ± s.e.m.). Significance was determined using two way analysis of variance (*p < 0.05). **c** After 20 weeks, weight loss was calculated based on the change from a mouse’s maximal weight when consuming the HFD compared to the final weight following the diet change (*n* = 5 mice; paired *t* test). **d** Inguinal mammary gland weight after 20 weeks on respective diets (*n* = 5 mice/group). **e** Mammary glands were stained for smooth muscle actin (SMA, red) and 4′6-diamidine-2′-phenylidole dihydrochloride (DAPI) (blue) using immunofluorescence and five images were scored per mouse (*n* = 5 mice/group). **f** Mammary glands were immunohistochemically stained for estrogen receptor (ER)α, and the percentage of ERα^+^ mammary epithelial cells/duct were calculated from the Con or HFDΔCon mice (*n* = 5 mice/group). Mammary epithelial cells from glands from the HFDΔCon or Con mice were plated in limiting dilution on an NIH 3T3 cell feeder layer on adherent plates (**g**), collagen gels (**h**) and as mammospheres on ultra-low attachment plates (**i**). Adherent colonies and collagen gels were quantified in duplicate, and primary and secondary generation mammospheres were quantified in triplicate (*n* = 5 mice/group). Bars represent mean ± s.d. Magnification bar = 100 μm. Avg, Average

Since we observed changes in mammary epithelial cell populations and progenitor activity in response to obesity, we hypothesized that these changes may be reversible with weight loss. To examine weight loss, 8-week-old C57Bl/6 mice were started on the control diet or HFD for 15 weeks. Mice in the HFD group significantly increased both body weight and weight gain within 15 weeks (Fig. 6a, b). At 15 weeks, the HFD was changed to the control diet and changes in body weight were examined. During the first week of diet change, all mice in the HFD group lost weight, with an average weight loss of 5.5 g ± 2.8 g (mean ± s.d.; Fig. 6b). The greatest weight loss was observed during the first week; however, after 5 weeks on the control diet, all mice weighed significantly less than when consuming the HFD (Fig. 6c). After 20 weeks on the diets, the inguinal mammary gland weight was not significantly different between the control and weight loss groups (Fig. 6d).

To assess changes in the mammary epithelial cell populations, we examined SMA and ERα expression within the tissues from both diet groups. In the weight loss group, SMA was continuous surrounding the mammary ducts, similar to findings observed in the glands from control mice (Fig. 6e). ERα expression levels were also not significantly different between the control and weight loss groups (Fig. 6f). These results suggest that weight loss altered the mammary epithelial cell populations to be consistent with the control mice. To examine the effects of weight loss on progenitor activity within the mammary epithelial cells, mammary glands from the control and weight loss group were dissociated and epithelial cells were plated at limiting dilution on adherent plates, on collagen gels and as mammospheres on ultra-low attachment plates. In all progenitor assays, there were no significant differences between the control and weight loss groups (Fig. 6g-i). Together, these results suggest that weight loss reverses the changes in mammary epithelial cell populations observed with obesity.

## Discussion

Obesity has divergent effects on breast cancer risk, depending on whether weight gain occurs early in life or following menopause. To understand how obesity alters normal breast tissue, potentially leading to increased risk of breast cancer, we examined the consequences of obesity in a well-characterized HFD mouse model and in human breast tissue samples from reduction mammoplasty surgery. Using these tissues, we identified global changes in both human and mouse epithelial cell populations and in mammary gland architecture that might lead to the observed changes in breast cancer risk over time.

Breast cancer can be divided into distinct subtypes based on gene expression profiling [62–64]. These divergent subtypes have been hypothesized to arise due to differences in mutations and distinct cells of origin within the breast (for review see [16, 65, 66]). Studies using targeted expression of oncogenes in the mammary epithelium have demonstrated that luminal lineage cells generate tumors that are more aggressive and heterogeneous than epithelial cells from the basal lineage [20, 67, 68], leading to the hypothesis that luminal progenitor cells are the cells of origin for the most common types of breast cancer [18, 19]. If breast cancers originate in distinct stem/progenitor cell populations, it also suggests that the risk of cancer development may be related to the size of the progenitor cell pool and its mitotic activity [17]. Our studies show that obesity significantly enhances luminal cells in mice and mature luminal and luminal progenitor cells in women. While postmenopausal women have an increased risk of developing ERα^+^ luminal breast cancers [24–26], both premenopausal and postmenopausal obese women also have an increased likelihood of being diagnosed with ERα^-^ tumors compared with lean women [29, 30]. These results suggest that obesity may enhance the risk of development of different subtypes of breast cancer through the expansion of luminal progenitor cells that may give rise to the most common types of breast cancer. Studies utilizing lineage tracing in the context of obesity will be necessary to more directly assess how changes in epithelial cell populations contribute to the formation of different tumor histological types.

Epidemiologic studies have suggested that increased ERα expression in breast epithelial cells increases breast cancer risk, particularly in postmenopausal women [69–71]. Our studies suggest that obesity enhances ERα^+^ cells in obese mice and in the breast tissue of premenopausal and postmenopausal women. Interestingly, while weight gain in premenopausal women mildly decreases breast cancer risk before menopause [9, 10], premenopausal weight gain enhances the risk of development of ERα^+^ breast tumors after menopause [72]. The cells of origin for ERα^+^ breast tumors are currently unclear, but tumors may originate in ERα^+^ progenitor cells. In both mice and humans, rare ERα^+^ cells have been identified that co-label with markers of proliferation [58–60, 73]. These ERα^+^ proliferating cells are rare in breast tissue from young women, but are enhanced with age [74, 75]. Our studies suggest that obesity also enhances ERα^+^ cells that co-label with markers of proliferation in both mice and humans. ERα^+^ epithelial cell populations have been shown to have limited progenitor activity within the mouse mammary gland [38, 76, 77]. Recent lineage tracing studies have demonstrated that ERα^+^ progenitor cells maintain the ERα^+^ luminal cell lineage [76, 78]. Examination of ERα^+^ progenitor cells in the context of obesity using these lineage tracing models could provide insight into how obesity alters ERα^+^ progenitor cells and their contribution to ERα^+^ breast tumors.

Progression from ductal carcinoma in situ (DCIS) to invasive ductal carcinoma is characterized by invasion of breast cancer cells into the surrounding tissue due to the physical breach of the basal/myoepithelial cell layer and underlying basement membrane [79]. Our studies have shown that within mammary glands from obese mice, basal/myoepithelial epithelial cell populations are reduced, and SMA^+^ basal/myoepithelial cells discontinuously surround luminal cells, compared with control mice. Given their central role in suppression of tumor progression and invasion (for review see [21–23]), discontinuity of the basal/myoepithelial layer under conditions of obesity may weaken local defenses, which could facilitate tumor invasion into the surrounding tissue once cancer arises. Obesity increases the risk of the development of invasive breast cancer in postmenopausal women [80], and loss of the protective basal/myoepithelial layer may contribute to this risk by allowing more rapid progression of DCIS to invasive breast tumors. Our results suggest that disease models to examine the impact of obesity-induced basal/myoepithelial cell loss on the progression of DCIS to invasive ductal carcinoma may be best addressed using transgenic mouse models of tumor progression or intraductal transplantation in obese mice to model human DCIS [81, 82].

Although postmenopausal obesity is a significant risk factor for breast cancer, epidemiological studies suggest that obesity during puberty decreases life-long breast cancer risk [10, 72, 83], and decreases the risk of developing benign breast disease [84]. Puberty is an important stage of growth in the normal mammary gland, with the elongation of ducts and secondary branches forming from terminal end buds to fill the mammary fat pad. When 3-week-old mice were fed a HFD, we observed significant reductions in ductal branching. Our results are consistent with a previous study demonstrating significant reductions in terminal end buds and ductal elongation in BALB/C and C57Bl/6 mice fed a HFD for 4 weeks [85]. Together, these studies suggest that obesity during puberty alters the architecture of the mammary gland, leading to reduced ductal development. Reduced mammary epithelial development may decrease breast cancer risk over a woman’s lifetime by limiting the total number of epithelial stem/progenitor cells that could accrue mutations and form malignant growths. However, the effects of obesity on breast development in the context of obesity during puberty have not been examined in humans, and how these changes might contribute to the decreased breast cancer risk in premenopausal women observed epidemiologically are unknown. Obesity has also been associated with alterations in the menstrual cycle and decreased ovulation in young women (for review see [86]). We did not observe significant differences in the length of the estrus cycle in lean and obese mice or significant changes in uterine weight, which might suggest imbalances in ovarian hormones. However, more subtle differences in the estrus cycle may be present, which we were unable to detect.

Although we have demonstrated that obesity leads to alterations in mammary epithelial cell populations and enhanced ERα expression, the mechanism underlying these observations may be complex. Leptin, which is secreted by adipocytes, is increased under conditions of obesity and has been shown to enhance ERα expression in both normal epithelial cells and breast cancer cell lines [87, 88]. Leptin has also been shown to stimulate breast stem cell self-renewal in mammosphere assays utilizing primary human breast epithelial cells [89]. In addition to enhanced leptin secretion, obesity increases the recruitment of inflammatory macrophages into mammary adipose tissue [35, 37, 90]. Chronic inflammation has been shown to result in increased aromatase expression within mammary tissue [90], and increased expression of cytokines, such as interleukin (IL)-6 and IL-8, which have been shown to regulate breast cancer stem cells [91]. Obesity may also alter transforming growth factor beta (TGFβ) release and bioavailability to mammary epithelial cells [92, 93]. TGFβ signaling inhibits the proliferation of ERα^+^ epithelial cells in mouse mammary glands [94, 95] and cultured human breast epithelial cells [96], and loss of TGFβ signaling may play a role in the observed increases in ERα^+^ proliferating cells in mammary tissue in obesity. These signaling pathways may act in concert or have divergent effects on separate epithelial cell populations within the luminal and basal/myoepithelial mammary epithelium.

Weight loss is an attractive intervention for reduction of breast cancer risk. Data from the Prospective Nurses’ Health Study suggests that weight loss after menopause is associated with lower incidence of breast cancer [97]. Additionally, a group of women who lost weight with bariatric surgery were shown to have reduced risk of breast cancer [98]. Our results suggest that in mice that lost weight, the percentage of ERα^+^ cells in normal mammary epithelium was similar to that in control mice. Additionally, there were no significant differences observed in any in vitro progenitor assays. These results suggest that weight loss may reverse the enrichment of progenitor cells induced by obesity, potentially decreasing the cells of origin for breast cancer to similar levels found in lean mice. We also observed increased continuity of the SMA^+^ basal/myoepithelial layer, which also suggests that the protective layer surrounding the luminal cells is regained with weight loss. Recent studies in postmenopausal obese women and in obese and overweight breast cancer survivors following treatment suggest that weight loss decreases serum levels of leptin, insulin, and estrogens [99, 100], and Ki67 expression in normal breast epithelial cells [100]. These results suggest that weight loss may reduce serum levels of growth factors that may influence progenitor activity within the breast epithelium. Although we observed reductions in progenitor activity with weight loss in mice, another study examining epigenetic changes in the mammary gland in obesity demonstrated that weight normalization did not reverse the effects of obesity on epigenetic reprogramming and inflammatory signals in the mammary gland microenvironment [101]. Further studies are necessary to understand how the timing and use of weight loss strategies may have maximal impact for the reduction of breast cancer risk in obese women.

## Conclusions

Obesity enhances the risk of breast cancer in postmenopausal women but decreases the risk of breast cancer in young women. Our data suggest that obesity increases luminal cells and ERα expression, reduces the total number of basal/myoepithelial cells, and enhances epithelial stem/progenitor activity in both mice and humans. These changes may contribute to lifetime risk of the development of ERα^+^ tumors in postmenopausal women. However, the changes observed in mammary epithelial cell populations induced by obesity were reversible with weight loss, suggesting that weight loss interventions in obese women may reduce breast cancer risk by returning ERα expression patterns and basal/myoepithelial cell numbers to normal levels. Our findings support further studies to examine how obesity-induced changes in stem/progenitor cells impact breast cancer risk and tumor histological types.

## Additional files

Additional file 1: Figure S1. High-fat diet (HFD) feeding increases mammary adipocyte size and inflammation. **A** C57Bl/6 female mice were fed a HFD or control diet (Con) starting at 8 weeks of age. Fat surrounding the uterus (gonadal fat) was weighed in Con and HFD mice (*n* = 7 mice/group). **B** Representative images of mammary glands from Con and HFD mice, stained with F4/80 to detect crown-like structures. **C** Quantification of adipocyte diameters from mammary glands of Con and HFD mice (*n* = 7 mice/group). **D** Quantification of crown-like structures from mammary glands of Con and HFD mice (*n* = 7 mice/group). No crown-like structures were observed in the mammary glands of Con mice. Bars represent mean ± s.d. Magnification bar = 100 μm. (PDF 230 kb)

Additional file 2: Figure S2. Flow cytometry and cytokeratin 14 immunofluorescence staining. **A** Representative contour plots to demonstrate gating for mouse mammary epithelial cell flow cytometry. Cells were gated in SSC and FSC to eliminate debris. Live cells were identified as negative for fixable viability dye eFluor 780. PE-conjugated stromal lineage markers, including CD31, CD45, and Ter119, were then removed from further analysis through gating. **B** Representative contour plots to demonstrate gating for human breast epithelial cell flow cytometry. Human breast epithelial cells were dissociated and lineage-depleted using bead-conjugated antibodies prior to analysis for flow cytometry as described in “Methods”. Cells were gated in SSC and FSC to eliminate debris, and live cells were identified as negative for fixable viability dye eFluor 780. **C** Cytokeratin (CK)14-positive cells (red) were detected in human reduction mammoplasty tissue using immunofluorescence. Nuclei were detected with DAPI (blue). CK14 expression was scored as described in “Methods” and graphed in relation to patient body mass index (BMI; n = 30). Magnification bar = 100 μm. (PDF 622 kb)

Additional file 3: Figure S3. Obesogenesis in response to a high-fat diet with respect to timing and mouse strain. **A** Eight-week-old C57Bl/6 mice (B6-8 wk) were fed a high-fat diet (HFD) or control diet (Con). Weight gain was determined for 17 weeks (*n* = 7 mice/group; mean ± s.e.m.). Differences were determined using two way ANOVA. Eight-week-old FVB/N female mice (FVB-8 wk) were fed a HFD or Con for 14 weeks (*n* = 5 mice/group). Body weight (**B**) and weight gain (**C**) were measured (mean ± s.e.m.). No differences were detected using two way ANOVA. **D** Inguinal gland weight from FVB-8 wk mice fed either HFD or Con. **E** Quantification of tertiary branching in whole mounts from HFD and Con inguinal mammary glands from FVB-8 wk mice. **F** Three-week-old C57Bl/6 mice (B6-3 wk) were fed a HFD or Con. Weight gain was determined for 17 weeks (*n* = 9 mice/group; mean ± s.e.m.). **G** Fat surrounding the uterus (gonadal fat) was weighed for B6-3 wk Con and Mice fed HFD (*n* = 9 mice/group). **H** Quantification of adipocyte diameters from mammary glands of B6-3 wk Con and HFD mice (n = 9 mice/group). **I** Quantification of crown-like structures (CLS) from mammary glands of B6-3 wk Con and HFD mice (*n* = 9 mice/group). No CLS were observed in the mammary glands of Con mice. **J** 3-week-old FVB/N mice (FVB-3 wk) were fed a HFD or Con diet. Weight gain was determined for 17 weeks (*n* = 9 mice/group; mean ± s.e.m.). **K** Gonadal fat was weighed for FVB-3 wk Con and HFD mice (*n* = 9 mice/group). **L** Quantification of adipocyte diameters from mammary glands of FVB-3 wk Con and HFD mice (*n* = 9 mice/group). **M** Quantification of CLS from mammary glands of FVB-3 wk Con and HFD mice (*n* = 9 mice/group). No CLS were observed in the mammary glands of Con mice. Bars represent mean ± s.d. (PDF 151 kb). (PDF 151 kb)

Additional file 4: Figure S4. Effect of obesity on estrus cycle in C57Bl/6 and FVB/N mice. **A** Quantification of average days in estrus and diestrus over 2 weeks using vaginal cytology from C57Bl/6 fed a high-fat diet (HFD) or control diet (Con) starting at 3 weeks of age (n = 9/group). **B** Quantification of average days in estrus and diestrus over 2 weeks using vaginal cytology from FVB/N mice fed a HFD or Con starting at 3 weeks of age (n = 9/group). **C** Uterine weights after 17 weeks on HFD or Con from C57Bl/6 mice fed at 3 weeks of age. **D** Uterine weights after 17 weeks on HFD or Con from FVB/N fed at 3 weeks of age. Bars represent mean ± s.d. (PDF 97 kb)

Additional file 5: Figure S5. Obesity reduces myoepithelial cells and enhances ERα-positive luminal cells in mice fed a high-fat diet (HFD) during puberty. **A** The 3-week-old C57Bl/6 female mice were started on a control diet (Con) or HFD for 17 weeks. Mammary epithelial cells were isolated and quantified using flow cytometry as described in “Methods”. The ratio of luminal cells (EpCAM^hi^CD49f^lo^) to basal cells (EpCAM^lo^CD49f^hi^) was quantified in three experiments (n = 6 mice). The percentage of luminal and basal cells was determined from total mammary lineage positive cells. **B** Myoepithelial cells were detected using immunofluorescence for SMA within the mammary glands of C57Bl/6 and FVB/N mice fed HFD or Con starting at 3 weeks of age. SMA continuity was scored as described in “Methods” (n = 3 images/gland; 5 mice/group). **C** ERα^+^ luminal cells were immunohistochemically detected within the mammary glands of C57Bl/6 and FVB/N mice fed the HFD or Con starting at 3 weeks of age (n = 5 images/gland; 5 mice/group). **D** Myoepithelial cells were detected using immunofluorescence for SMA within the mammary glands of FVB/N mice fed the HFD or Con starting at 8 weeks of age. SMA continuity was scored as described in “Methods” (n = 3 images/gland; 5 mice/group). **E** ERα^+^ luminal cells were immunohistochemically detected within the mammary glands of FVB/N mice fed the HFD or Con starting at 8 weeks of age (n = 5 images/gland; 5 mice/group). Bars represent mean ± s.d. (PDF 107 kb)

## Abbreviations

BMI: Body mass index
BP: Basal progenitors
BrdU: 5-Bromo-2′-deoxyuridine
BSA: Bovine serum albumin
CK: Cytokeratin
Con: Control diet
DAB: 3,3-Diaminobenzidine
DAPI: 4′6-Diamidine-2′-phenylidole dihydrochloride
DCIS: Ductal carcinoma in situ
DMEM: Dulbecco’s modified Eagle’s medium
EGF: Epidermal growth factor
ERα: Estrogen receptor alpha
HFD: High-fat diet
HRP: Horseradish peroxidase
IF: Immunofluorescence
IGF-1: Insulin-like growth factor-1
IHC: Immunohistochemical analysis
IL: Interleukin
IRB: Institutional review board
LP: Luminal progenitor
MB: Mature basal
ML: Mature luminal
PBS: Phosphate-buffered saline
PE: Phycoerythrin
s.d.: Standard deviation
SMA: Smooth muscle actin
TGFβ: Transforming growth factor beta
