# Supplementary figures and images for: Obesity reversibly depletes the basal cell population and enhances mammary epithelial cell estrogen receptor alpha expression and progenitor activity
Source: Breast Cancer Res. 2017 Nov 29;19:128. doi: 10.1186/s13058-017-0921-7 (PMC5707907; doi:10.1186/s13058-017-0921-7)

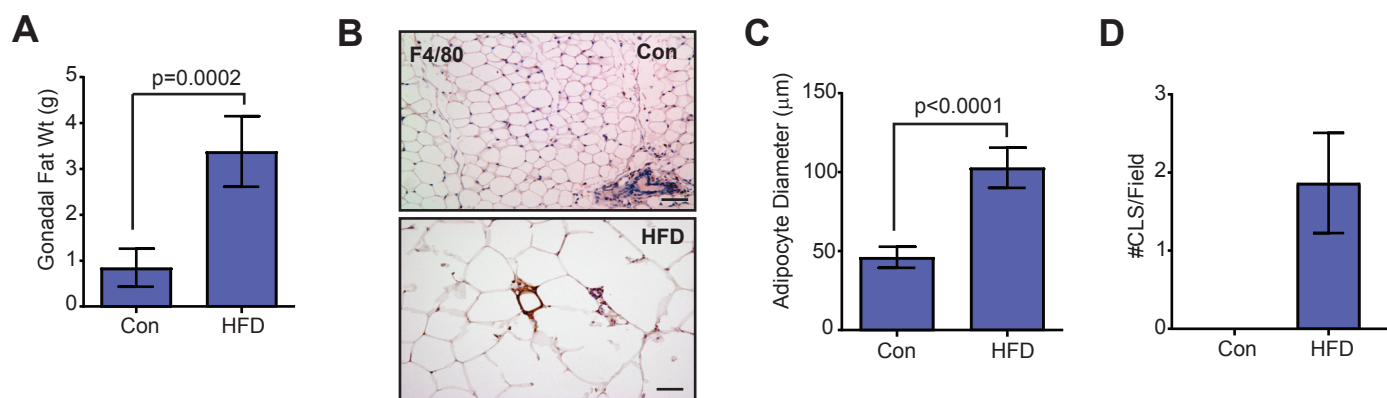

Supplement: Supplementary file 1 — High-fat diet (HFD) feeding increases mammary adipocyte size and inflammation. A C57Bl/6 female mice were fed a HFD or control diet (Con) starting at 8 weeks of age. Fat surrounding the uterus (gonadal fat) was weighed in Con and HFD mice (n = 7 mice/group). B Representative images of mammary glands from Con and HFD mice, stained with F4/80 to detect crown-like structures. C Quantification of adipocyte diameters from mammary glands of Con and HFD mice (n = 7 mice/group). D Quantification of crown-like structures from mammary glands of Con and HFD mice (n = 7 mice/group). No crown-like structures were observed in the mammary glands of Con mice. Bars represent mean ± s.d. Magnification bar = 100 μm. (PDF 230 kb) [file 13058_2017_921_MOESM1_ESM.pdf]

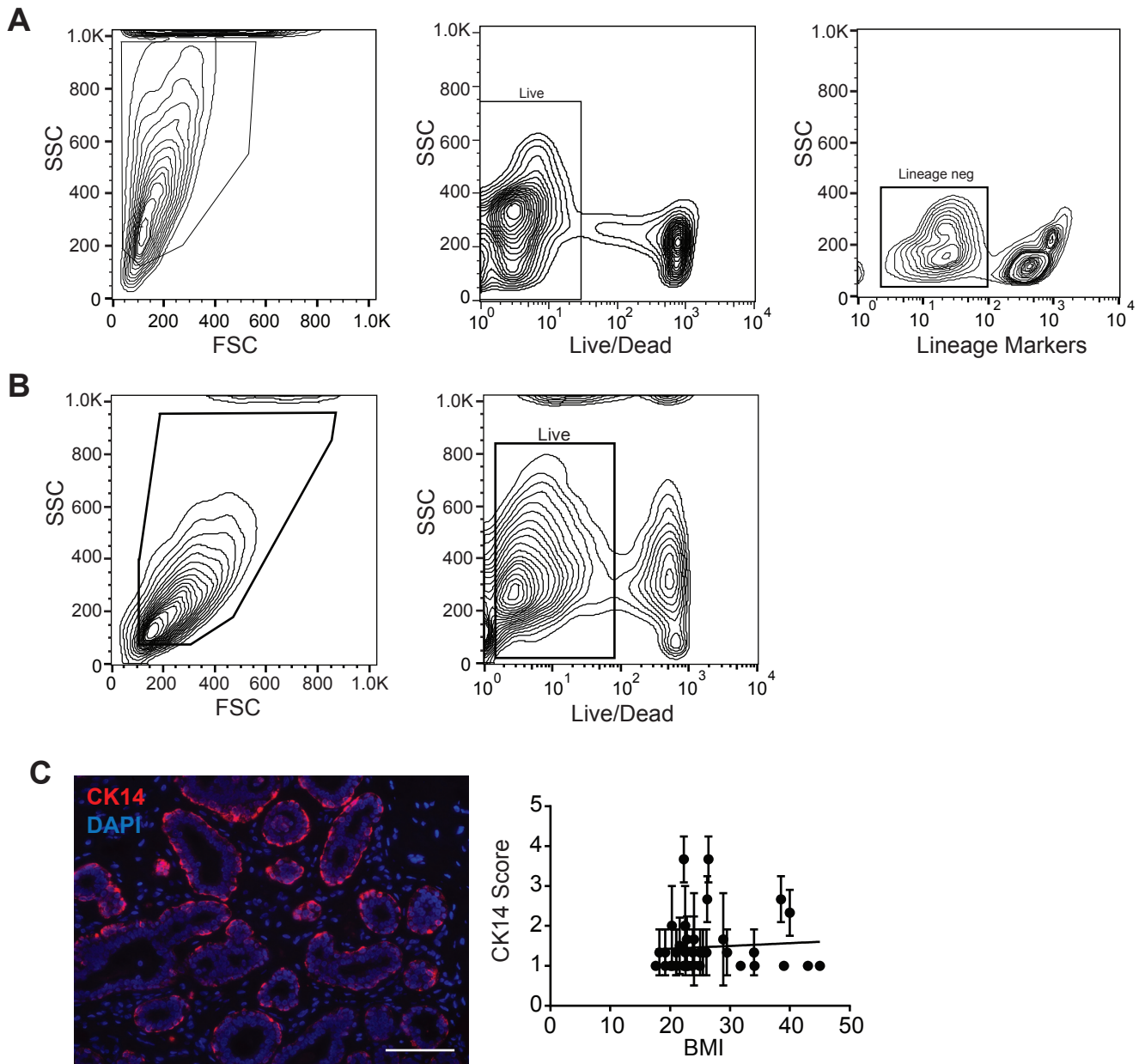

Supplement: Supplementary file 2 — Flow cytometry and cytokeratin 14 immunofluorescence staining. A Representative contour plots to demonstrate gating for mouse mammary epithelial cell flow cytometry. Cells were gated in SSC and FSC to eliminate debris. Live cells were identified as negative for fixable viability dye eFluor 780. PE-conjugated stromal lineage markers, including CD31, CD45, and Ter119, were then removed from further analysis through gating. B Representative contour plots to demonstrate gating for human breast epithelial cell flow cytometry. Human breast epithelial cells were dissociated and lineage-depleted using bead-conjugated antibodies prior to analysis for flow cytometry as described in “Methods”. Cells were gated in SSC and FSC to eliminate debris, and live cells were identified as negative for fixable viability dye eFluor 780. C Cytokeratin (CK)14-positive cells (red) were detected in human reduction mammoplasty tissue using immunofluorescence. Nuclei were detected with DAPI (blue). CK14 expression was scored as described in “Methods” and graphed in relation to patient body mass index (BMI; n = 30). Magnification bar = 100 μm. (PDF 622 kb) [file 13058_2017_921_MOESM2_ESM.pdf]

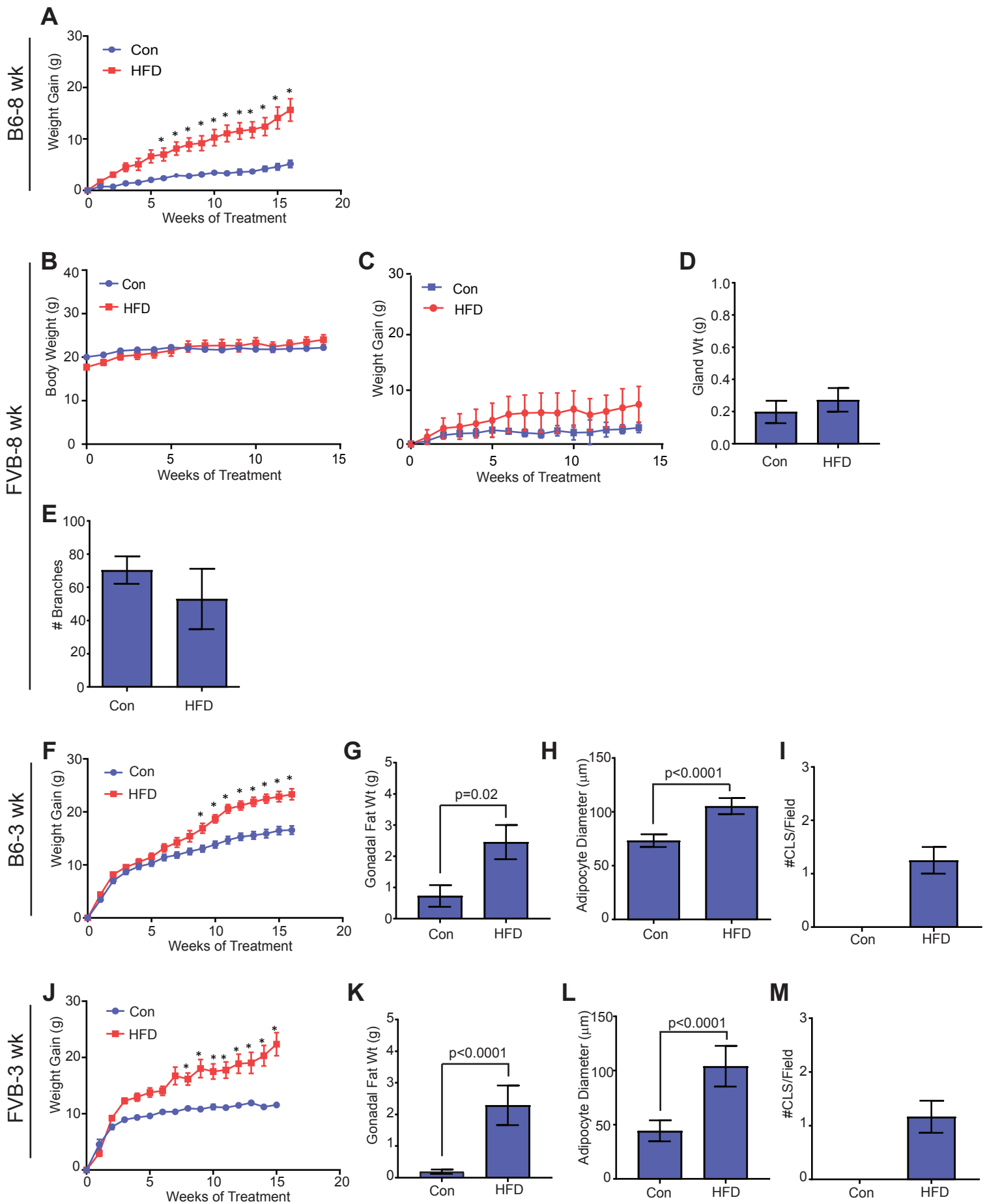

Supplement: Supplementary file 3 — Obesogenesis in response to a high-fat diet with respect to timing and mouse strain. A Eight-week-old C57Bl/6 mice (B6-8 wk) were fed a high-fat diet (HFD) or control diet (Con). Weight gain was determined for 17 weeks (n = 7 mice/group; mean ± s.e.m.). Differences were determined using two way ANOVA. Eight-week-old FVB/N female mice (FVB-8 wk) were fed a HFD or Con for 14 weeks (n = 5 mice/group). Body weight (B) and weight gain (C) were measured (mean ± s.e.m.). No differences were detected using two way ANOVA. D Inguinal gland weight from FVB-8 wk mice fed either HFD or Con. E Quantification of tertiary branching in whole mounts from HFD and Con inguinal mammary glands from FVB-8 wk mice. F Three-week-old C57Bl/6 mice (B6-3 wk) were fed a HFD or Con. Weight gain was determined for 17 weeks (n = 9 mice/group; mean ± s.e.m.). G Fat surrounding the uterus (gonadal fat) was weighed for B6-3 wk Con and Mice fed HFD (n = 9 mice/group). H Quantification of adipocyte diameters from mammary glands of B6-3 wk Con and HFD mice (n = 9 mice/group). I Quantification of crown-like structures (CLS) from mammary glands of B6-3 wk Con and HFD mice (n = 9 mice/group). No CLS were observed in the mammary glands of Con mice. J 3-week-old FVB/N mice (FVB-3 wk) were fed a HFD or Con diet. Weight gain was determined for 17 weeks (n = 9 mice/group; mean ± s.e.m.). K Gonadal fat was weighed for FVB-3 wk Con and HFD mice (n = 9 mice/group). L Quantification of adipocyte diameters from mammary glands of FVB-3 wk Con and HFD mice (n = 9 mice/group). M Quantification of CLS from mammary glands of FVB-3 wk Con and HFD mice (n = 9 mice/group). No CLS were observed in the mammary glands of Con mice. Bars represent mean ± s.d. (PDF 151 kb). (PDF 151 kb) [file 13058_2017_921_MOESM3_ESM.pdf]

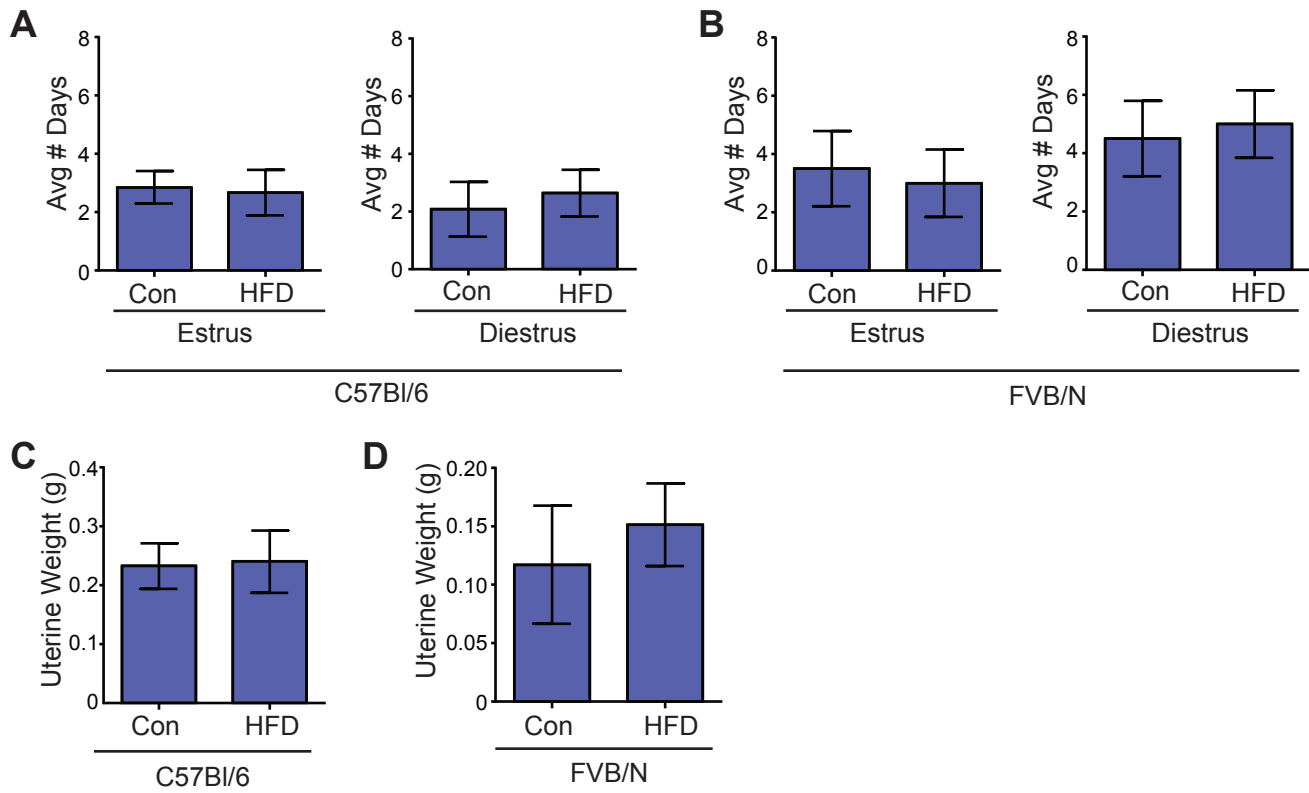

Supplement: Supplementary file 4 — Effect of obesity on estrus cycle in C57Bl/6 and FVB/N mice. A Quantification of average days in estrus and diestrus over 2 weeks using vaginal cytology from C57Bl/6 fed a high-fat diet (HFD) or control diet (Con) starting at 3 weeks of age (n = 9/group). B Quantification of average days in estrus and diestrus over 2 weeks using vaginal cytology from FVB/N mice fed a HFD or Con starting at 3 weeks of age (n = 9/group). C Uterine weights after 17 weeks on HFD or Con from C57Bl/6 mice fed at 3 weeks of age. D Uterine weights after 17 weeks on HFD or Con from FVB/N fed at 3 weeks of age. Bars represent mean ± s.d. (PDF 97 kb) [file 13058_2017_921_MOESM4_ESM.pdf]

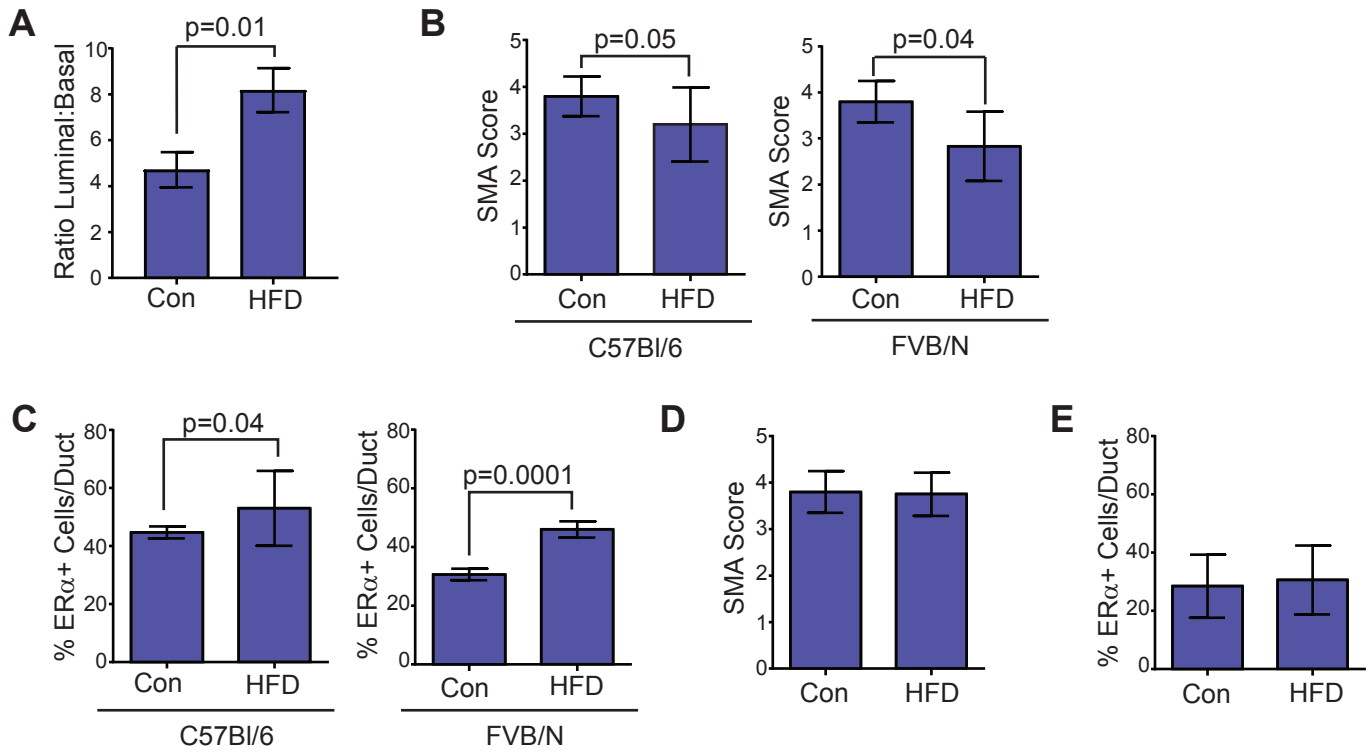

Supplement: Supplementary file 5 — Obesity reduces myoepithelial cells and enhances ERα-positive luminal cells in mice fed a high-fat diet (HFD) during puberty. A The 3-week-old C57Bl/6 female mice were started on a control diet (Con) or HFD for 17 weeks. Mammary epithelial cells were isolated and quantified using flow cytometry as described in “Methods”. The ratio of luminal cells (EpCAMhiCD49flo) to basal cells (EpCAMloCD49fhi) was quantified in three experiments (n = 6 mice). The percentage of luminal and basal cells was determined from total mammary lineage positive cells. B Myoepithelial cells were detected using immunofluorescence for SMA within the mammary glands of C57Bl/6 and FVB/N mice fed HFD or Con starting at 3 weeks of age. SMA continuity was scored as described in “Methods” (n = 3 images/gland; 5 mice/group). C ERα+ luminal cells were immunohistochemically detected within the mammary glands of C57Bl/6 and FVB/N mice fed the HFD or Con starting at 3 weeks of age (n = 5 images/gland; 5 mice/group). D Myoepithelial cells were detected using immunofluorescence for SMA within the mammary glands of FVB/N mice fed the HFD or Con starting at 8 weeks of age. SMA continuity was scored as described in “Methods” (n = 3 images/gland; 5 mice/group). E ERα+ luminal cells were immunohistochemically detected within the mammary glands of FVB/N mice fed the HFD or Con starting at 8 weeks of age (n = 5 images/gland; 5 mice/group). Bars represent mean ± s.d. (PDF 107 kb) [file 13058_2017_921_MOESM5_ESM.pdf]
